# How Is Bovine Genital Leptospirosis Diagnosed Under Field Conditions?

**DOI:** 10.3390/ani15030443

**Published:** 2025-02-06

**Authors:** Juliana Pedrosa, Julia Mendes, José Zambrano, Filipe Aníbal Carvalho-Costa, Maria Isabel Nogueira Di Azevedo, Luiza Aymée, Walter Lilenbaum

**Affiliations:** 1 Laboratory of Veterinary Bacteriology, Biomedical Institute of Fluminense Federal University, Niterói 24220-900, RJ, Brazil; juliana_pedrosa@id.uff.br (J.P.); isabeldiazevedo@gmail.com (M.I.N.D.A.); luizaaymeeps@gmail.com (L.A.); 2 Zambrano Herd Consulting Corporation, Belo Horizonte 30421-300, MG, Brazil; 3 Laboratory of Epidemiology and Molecular Systematics, Oswaldo Cruz Institute, Oswaldo Cruz Foundation, Rio de Janeiro 21040-90, RJ, Brazil

**Keywords:** bovine, leptospirosis, diagnosis, microscopic agglutination testing, PCR

## Abstract

Bovine genital leptospirosis (BGL) is a chronic reproductive disease in cattle, often causing significant economic losses, and is commonly associated with leptospiral strains belonging to the Sejroe serogroup. A two-step protocol was recommended, based on serological screening of the herds followed by an individual diagnosis with PCR of a genital sample. Although proposed, it has not been commonly applied under field conditions, leading to frustrating outcomes in disease control. In that context, the present study aimed to demonstrate the viability of that two-step protocol under field conditions for diagnosing BGL in eight herds with reproductive disorders. Blood samples were collected from 440 cows for serology. In addition, 304 cervicovaginal mucus (CVM) samples were collected for *lip*L32-PCR and 11 samples were sequenced of the *sec*Y gene. All herds showed high seroreactivity, mainly against the serogroup Sejroe. In addition, 113 of the 304 CVM samples (37.2%) were PCR-positive. DNA sequencing of 11 positive samples based on the *sec*Y gene revealed maximum identity (100%) with *L. interrogans* species. This study found CVM sampling quick and easy, making it practical for field use. Overall, the results support the two-step protocol serological screening followed by CVM-PCR testing as an efficient and reliable method for diagnosing BGL in herds with reproductive disorders in field conditions.

## Simple Summary

Bovine leptospirosis is an important reproductive disease. This study evaluated the effectiveness of a two-step protocol for diagnosing bovine genital leptospirosis (BGL) in eight herds with reproductive disorders. The protocol involved serological screening followed by PCR testing of cervicovaginal mucus (CVM) samples. Blood samples from 440 cows were collected for serology, while 304 CVM samples were tested using *lip*L32-PCR and 11 positive samples were sequenced for *sec*Y gene. Results showed high seroreactivity against the Sejroe serogroup, and 37.2% of the CVM samples were PCR-positive. DNA sequencing revealed maximum identity (100%) with *L. interrogans* species. This study found CVM sampling quick and easy, making it a practical diagnostic tool under field conditions. Overall, the findings support the two-step protocol as an efficient and reliable method for diagnosing BGL in cattle with reproductive issues.

## 1. Introduction

Leptospirosis is an environmentally transmitted disease and a major zoonotic cause of morbidity and mortality worldwide, representing a significant One Health burden in humans, domestic animals, and livestock [1]. The disease is caused by spirochetes of the genus *Leptospira*, with 64 known genomospecies classified phylogenetically into two large clades: “Pathogens” (37 genospecies), containing all species responsible for infections in humans and/or animals, and “Saprophytes” (27 genomospecies), which include environmental species for which the virulence status has not been proven [2]. Pathogenic leptospires can establish chronic carriage in the renal tubules of reservoir hosts, which are critical for the persistence and transmission of the disease in the environment.

Leptospires can be classified by genetic or phenotypic features [3]. Regarding the serological classification, the leptospiral strains are divided based on the characteristics of the lipopolysaccharides (LPS) of their outer membrane [4]. In this classification, strains are classified into approximately 300 serovars and grouped into 24 serogroups according to the homogeneity of the LPS [5]. Pathogenic and non-pathogenic serovars can belong to the same species; therefore, the use of both classifications for determining a strain is well established, since genetic heterogeneity within the same serovar has been demonstrated [4].

The disease can occur in diverse epidemiological settings due to the large spectrum of wild and domestic mammals that serve as reservoirs. The high mobility conferred by leptospiral periplasmic flagella allows the bacteria to invade the host organism by active penetration through injured skin or intact mucosa and colonize the tissue [3]. Those animals harbor and excrete the agent from their renal tubules, contaminating water and soil and serving as a source of spillover infections to humans and animals. In humans, as well as in dogs, the disease is acute, leading to a severe syndrome characterized by icterus, hemorrhages, and kidney failure, frequently leading to death [6].

Infections in cattle occur primarily through direct contact with urine or indirectly through contact with contaminated water or fomites such as drinkers and feeders. This transmission mechanism is particularly common for incidental infections, where other host species such as rodents act as reservoirs of *Leptospira* [6]. However, for infections caused by strains specifically adapted to cattle, direct contact between cattle is a crucial factor in the spread of the disease. In such cases, sexual transmission should also be considered, as it can play a significant role in the spread of *Leptospira* within herds [7]. Therefore, disease control involves addressing both environmental and direct animal-to-animal transmission routes.

In cattle, the disease usually manifests as a chronic condition that leads to various reproductive disorders, including repeated estrus, embryonic death, stillbirths, and abortions. Embryonic mortality is the major symptom of bovine leptospirosis and is classified as early embryonic death (EED) when it occurs until 28 days after fertilization, while late embryonic death (LED) includes a period of 29 to 45 days [8]. These reproductive failures significantly interfere with herd productivity, leading to significant economic losses. In addition, the disease’s long-term impact on herd fertility can reduce the overall production of milk and meat, increasing veterinary costs and decreasing profitability for farmers [9,10].

Those manifestations have often been associated with long-term genital infection by leptospires, named bovine genital leptospirosis (BGL) [11]. Other signs that are a consequence of leptospirosis infection are abortions, stillbirths, or the birth of weak calves [12]. Abortions have been widely associated with bovine leptospirosis, as they are linked to incidental strains when they occur as an outbreak or to adapted strains when they occur endemically [9].

The interaction between the affected host and the infecting strain may lead to different clinical aspects in cattle. More severe abortion clinical signs such as fever, jaundice, and epizootic abortions are infrequent in cattle and usually occur as outbreaks; they are associated with incidental serovars such as Pomona, Grippotyphosa, and Icterohaemorrhagiae [13]. Conversely, adapted serovars from Sejroe serogroups, such as Hardjo genotypes (*L. interrogans* Hardjoprajitno and *L. borgpetersenii* Hardjobovis), as well as Guaricura (*L. santarosai*), frequently identified in South American cattle [14], lead to a subclinical and silent disease in cattle. Although the dynamics of genital infection, adaptability to the host, and the effects of infection in cattle by serovar Guaricura are not yet completely understood, it is known that, together with Hardjo strains, they are the major agents of subfertility or even infertility in cattle, which makes the diagnosis difficult to determine [14,15].

Transmission of leptospires generally occurs through the release of bacteria in the urine and other fluids of the infected animal. Strains belonging to the Sejroe serogroup can be transmitted from cattle to cattle, and not only through the contaminated environment. Considering the reports of the presence of leptospires in both the male and female reproductive tract [7,16], sexual transmission in cattle is suggested, both from males to females and from females to males [7,17]. In females, besides the kidneys, leptospires have been detected in different reproductive organs, such as the uterus [15], oviduct, and ovaries [18,19]. In the male, leptospires or their DNA was detected in the semen of bulls and rams [17,20], vesicular gland, epididymis, and vas deferens of bucks [21] and boars [22].

Furthermore, many chronically infected animals may remain seronegative or have low titers in the Microscopic Agglutination Test (MAT), due to the adaptability of the bacteria to the host and the ability to evade the immune system, making them difficult to detect [23]. These animals may act as genital carriers of the infection for long periods, silently spreading the disease within the herd. As a result, many carriers remain undiagnosed and, consequently, untreated, impairing herd productivity. This persistent problem contributes to the common observation that control programs are often frustrating and ineffective [11].

Currently, the diagnosis of leptospirosis is based on serology, mainly the Microscopic Agglutination Test (MAT), as well as direct methods, such as culture and/or polymerase chain reaction (PCR) [24]. Although bacterial culture is commonly used to diagnose bacterial diseases and is the gold standard for leptospirosis diagnosis [23], it is not recommended for diagnostic purposes, as leptospires are fastidious bacteria that are difficult to grow. Therefore, culturing has low sensitivity to detecting infected animals [25]. When applied, PCR is usually conducted on urine instead of genital samples, reducing its diagnostic value for BGL. Therefore, serology still appears as the most available and inexpensive tool for diagnosing bovine leptospirosis on a large scale; unfortunately, as frequently reported, although useful and reliable for detecting infected herds, it cannot detect carriers, which are frequently seronegative or present low titers. Detection of carriers is essential for the specific treatment of the disease.

When BGL was first described, the authors suggested [11], based on theoretical considerations, that a two-step protocol might be necessary for its diagnosis. This protocol involved an initial serological screening of herds, followed by the individual detection of carriers using PCR from genital samples, specifically cervicovaginal mucus (CVM). More recently, a small-scale, region-specific study conducted by our group applied this protocol, showing encouraging results [26]. However, there is still a need to evaluate its feasibility on a larger scale, with a larger number of herds and animals in different regions. Therefore, the present study aimed to evaluate and validate the practicality and effectiveness of a two-step diagnostic protocol (MAT + PCR) under field conditions on a larger scale in different regions. By applying this approach to various herds experiencing reproductive disorders, this study sought to validate its broader applicability and reliability for diagnosing BGL in the field, potentially improving disease control efforts.

## 2. Materials and Methods

This study was conducted using samples submitted and collected for leptospirosis diagnosis at the Laboratory of Veterinary Bacteriology of the Fluminense Federal University, Niterói, Brazil. Blood and CVM samples from six herds (A, B, C, D, E, and F) were sampled by a practitioner and sent to the Laboratory of Veterinary Bacteriology for a routine diagnosis of leptospirosis, as part of their herd health program. The samples of the other two herds (G and H) were collected as a part of a study, conducted under protocol number 9527220222 in the Ethical Committee of Animal Use of the Federal Fluminense University. The laboratory performed serological screening tests (MAT) and PCR to detect leptospirosis infection in herds. Producers provided information about ongoing reproductive problems in herds and vaccinations.

### 2.1. Study Design

Eight herds with a history of chronic infertility, repeated heats, and episodic abortions were studied, all vaccinated more than four months ago against leptospirosis. Despite vaccination, these herds showed poor reproductive performance, with cows failing to conceive after three attempts at artificial insemination. To assess the infection status, serology was performed using the Microscopic Agglutination Test (MAT), and herds were considered infected if more than 10% of the animals showed seroreactivity against Sejroe strains [12]. After this screening, cervicovaginal mucus (CVM) samples were collected from cows with reproductive disorders to detect genital carriers.

### 2.2. Samples

The herds were located in the southeast region (São Paulo, Minas Gerais, and Rio de Janeiro states) of Brazil (Table 1). A total of 440 adult cows, made up of 10% sampling from each of the different herds, were selected based on the reproductive disorder criterion. This criterion was based on cows in the herd that underwent three AI attempts without a successful pregnancy. Blood samples were collected from all 440 cows for microscopic agglutination testing, and 304 cervicovaginal mucus (CVM) samples were collected for *lip*L32-PCR. It was not possible to collect the CVM from all cows due to logistical problems such as the impossibility of collection and infrastructural problems.

CVM was collected using cytology brushes (Kolplast) in a cytological device (Botupharma). The collection process was carried out in the region anterior to the urinary meatus, specifically, in the vaginal fornix, using a sterile cytological brush, that is, cervicovaginal mucus [11]. All collected CVM and sera were immediately refrigerated and transported to the Laboratory of Veterinary Bacteriology of Federal Fluminense University, Brazil for testing.

### 2.3. Serology

Serum samples were tested for antibodies against leptospirosis by MAT using an antigen panel composed of the following reference strains: *Leptospira interrogans* serovar Copenhageni (strain M20), Hardjoprajitno (strain OMS), Pomona (strain Pomona), Icterohaemorrhagiae (strain Verdun), Canicola (strain Hund Utrecht), Bratislava (strain Jez Bratislava), Australis (strain Ballico), Autumnalis (strain Akiyami A), and Grippotyphosa (strain Moskva V), *Leptospira borgpetersenii* serovar Hardjobovis (strain Sponselee), all originating from Institut Pasteur, plus *Leptospira santarosai* serovar Guaricura (strain FV52), a local strain recovered from the vaginal fluid of a cow, originated from Universidade de São Paulo. The selected strains for the antigen panel are known to be prevalent in cattle in Brazil [27] and represent seven serogroups: Australis, Autumnalis, Icterohaemorrhagiae, Canicola, Sejroe, Pomona, and Grippotyphosa. The reactivity of the sera was evaluated at titers of 100. A serogroup was classified as predominant in a herd when at least 50% of all the seroreactive sera presented a reaction against it.

### 2.4. Molecular Analysis

DNA was extracted from CVM samples (*n* = 304) using the DNeasy Blood & Tissue Kit (Qiagen, Hilden, Germany) according to the manufacturer’s instructions. PCR was performed targeting the *lip*L*32* gene for *Leptospira* spp. detection using the primers and conditions described by Hammond et al. [28]. For each set of samples, ultrapure water was used as the negative control in all reactions, and 10 fg of DNA extracted from *L. interrogans* serovar Copenhageni (Fiocruz L1-130) was used as the positive control. PCR products were analyzed by electrophoresis in 1.5–2% agarose gel and visualized under ultraviolet light after gel red staining. Platinum Taq DNA Polymerase (Invitrogen) was used for the reactions.

Genotyping was performed using the *sec*Y locus, a genetic marker with great discriminatory power [29]. A nested PCR was conducted according to Grillová et al., 2020 [30]. The Platinum Taq DNA Polymerase (Invitrogen, São Paulo, Brazil) was used for all reactions. The amplicons were purified with the PCR Clean-Up System Kit (Promega, Madison, WI, USA) according to the manufacturer’s instructions and intended for sequencing. PCR products were directly sequenced in both directions using BigDye Terminator 3.1 Cycle Sequencing Kit (Life Technologies, Foster City, CA, USA) on ABI 3730XL Genetic Analyzer (Life Technologies, Carlsbad, CA, USA) in PDTIS/Fiocruz genomic platform RPT01A. BioEdit v 5.0.9 was used to edit the sequences. The Basic Local Alignment Search Tool (BLAST-NCBI, https://blast.ncbi.nlm.nih.gov/Blast.cgi, accessed on 19 december 2024) was used to identify the *Leptospira* species based on nucleotide identity.

### 2.5. Statistics

A descriptive analysis (means and confidence interval (CI) of 95%) was performed regarding the frequencies of positive results in MAT and in PCR of each animal. The chi-square of the goodness-of-fit test was performed to compare MAT and PCR of CVM results to diagnose the infected animals. Additionally, ƙ (*Kappa*) index was estimated for MAT using the CVM-PCR as standard.

## 3. Results

The serology results showed that of the 440 tested sera, 337 (76.6%) were seroreactive (Table 1). The most frequent serogroup was Sejroe, which was predominant in 6/8 herds, followed by Icterohaemorrhagiae and Australis (one herd each). Regarding the molecular analysis, PCR showed that, out of the 304 CVM samples, 113 (39.2%) were positive for leptospiral DNA (Table 1).

**Table 1:** Results of the Microscopic Agglutination Test (MAT) of sera and polymerase chain. Reaction (PCR) of cervicovaginal mucus to diagnose bovine genital leptospirosis in cows from eight herds of Southeast Brazil presenting poor reproductive performance.

Herd	Sera (*n*)	MAT ≥ 100	Predominant Serogroup	CVM (*n*)	PCR Results	Sequenced Samples
A	40	26 (65%)	Sejroe	14	3 (21.4%)	3
B	59	56 (94.9%)	Australis	59	24 (40.7%)	
C	140	117 (83.5%)	Sejroe	70	14 (20%)	3
D	39	21 (53.8%)	Sejroe	23	11 (47.8%)	1
E	17	8 (47%)	Sejroe	17	10 (58.8%)	
F	60	37 (61.7%)	Icterohaemorrhagiae	36	12 (33.3%)	
G	63	54 (85.7%)	Sejroe	63	23 (36.5%)	4
H	22	18 (81.8%)	Sejroe	22	16 (72.7%)	1
**Total**	**440**	**337 (76.6%)**		**304**	**113 (37.2%)**	

All PCR-positive cows presented reproductive disorders such as subfertility, repeated estrus, embryonic death, and/or stillbirths. The positive results on PCR varied among the herds from 20% to 72.7%, with a mean of 41.4% (CI95%: 24.6–53.1%). In the statistical analysis, the chi-square test revealed a significant difference between the MAT and PCR results (*p* < 0.00001). The Kappa index, which measures the agreement between the two tests, was calculated at 0.46.

It was possible to sequence 11 *sec*Y amplicons, and all were identified as *L. interrogans*, with maximum identity (100%) with *L. interrogans* strain UF24 (MT270428). Sequences from the present study were deposited in GenBank under accession numbers PQ039544-PQ039551.

## 4. Discussion

Bovine genital leptospirosis is a chronic disease that leads to economic hazards, mainly due to reproductive losses. The real economic impact of BGL, i.e., the chronic form of the disease, which is mainly represented by embryonic mortality and subfertility, has never been calculated. However, the economic impact of bovine leptospirosis in its acute form, i.e., outbreaks mainly represented by abortions, has been estimated at $97 to $2611 per abortion [31]. Furthermore, an outbreak can lead to an annual risk of up to 150 thousand dollars, including abortions and costs of preventive measures, which is what occurred in Argentina [32]. Losses resulting from the reduction in productive and reproductive performance caused by leptospirosis were determined to be around 84% of the gross margin per liter of milk sold [33].

Due to the silent aspect of chronic disease, it is not as visible as abortion outbreaks, so its diagnosis can be challenging, which contributes to the underdiagnosis and the frustrating outcomes of control programs. Herein, we could validate the suggested protocol of MAT + PCR to diagnose BGL under field conditions in all the studied herds. In addition, a remarkable number of genital carriers presenting reproductive failures was identified (39.2%), reinforcing the role of BGL as an important reproductive disease in cattle [7,12]. Importantly, none of the studied herds/cows had presented with abortions or acute signs and would have remained underdiagnosed if the two-step protocol had not been adopted.

The serology by MAT is performed to diagnose leptospirosis on a worldwide basis [34,35] and it is the less onerous tool to perform several samples from a herd; besides that, it also can provide an epidemiological overview and indicate the infecting serogroup [27]. Herein, in most of the herds, the animals were seroreactive against the Sejroe serogroup, which is adapted to bovine. It is not a surprising outcome since Sejroe strains are known as the main agents of BGL [11] and have frequently been associated with reproductive failures [15,36]. Other detected serogroups were Icterohaemorrhagiae and Australis, which are incidental to cattle and adapted to other animal hosts [13]. Conversely to Sejroe strains, known as a cause of the subclinical and chronic reproductive disease in cattle, the incidental strains are commonly associated with abortion outbreaks, which were not observed in this study.

One of MAT’s main limitations is the impossibility of distinguishing vaccinal antibodies from infection [37]. Evidence reveals that vaccine antibody titers can remain high 2 to 4 months after application [38]. In addition, it has been acknowledged that MAT results are only reliable at the herd level [23] but do not predict individual infection in cattle [26]. In addition, many chronic genital carriers present with low titers or even seronegative, due to the adaptability of the bacterium to the host and the ability to evade the immune system. For those reasons, although MAT is important as a screening test in a herd, it must be complemented with molecular approaches to detect genital carriers at the individual level. In tropical countries the climatic characteristics, such as the high frequency of rainfall, warm temperatures, and soil pH, are favorable to the survival of the bacteria and for the preservation of its pathogenicity for long periods [24,39].

However, it is possible to observe seroreactivity for *Leptospira* in different regions. A study conducted in Uruguay showed an overall seroprevalence of *Leptospira* of 27.8% at the animal level, contrasting to 86.92% at the herd level, with a predominance of Sejroe and Pomona serogroups [40]. The same was reported in South Africa where the seroprevalence in the cattle sampled was 27.6%, with predominance of the serogroup Sejroe (38.2%) [41]. A study conducted in Egypt with cattle presenting clinical manifestations demonstrated that 39.3% of the sera evaluated were positive for antibodies against *Leptospira* [42].

Thus, in our study, we analyzed the agreement between the two tests (MAT and CVM-PCR) and observed that they present a moderate agreement, according to Fleiss’ [43] criteria. This indicates that they detect different aspects of leptospiral infection, and reinforces that MAT is not adequate to be used alone.

As direct methods of detection of the agents, the gold standard is certainly culturing the bacterium and obtaining pure isolates for genetic and serological characterization [23]. However, leptospires are very exigent about the culture media and are slow-growing and fastidious bacteria; thus, culturing presents low sensitivity for effective diagnosis, and is thus unfeasible for routine diagnosis under field conditions [25].

In that context, molecular techniques such as PCR and qPCR present high sensitivity and specificity for diagnosing leptospirosis [24]. These techniques can be performed on various clinical samples, such as urine, cervical–genital mucus, endometrial biopsy, semen, fetal tissues, and placenta [7,26].

The application of PCR for diagnosing BGL under field conditions has been conducted for a few years, with good results. Given that, PCR has become a crucial tool for BGL diagnosis, due to its high sensitivity [24]. In a study conducted in the Brazilian caatinga biome, 73.8% of the tested animals presented at least one organ/fluid positive for *Leptospira* spp. DNA [7]. Another study conducted in Colombia showed 37% positivity in cattle urine samples and demonstrated that PCR as a leptospirosis diagnostic technique was 100% sensitive and 99% specific compared to microbiological culture [44].

Historically, urine has been the standard sample for diagnosing leptospirosis in all animal species for culture and PCR. Furthermore, this sample has also been of interest in studies involving the application of control measures (treatment and vaccination) for bovine leptospirosis [45,46]. Testing urine samples of humans, dogs, and horses presenting renal manifestations during the course of the disease becomes necessary [3]. However, in bovine leptospirosis, kidney disease is uncommon and the recommendation to use urine is quite paradoxical, given that infected cattle commonly present reproductive failures [11]. Despite this, few studies focused on genital samples to investigate leptospiral infections in cattle, making this disease underdiagnosed.

A recent study by our group compared the use of genital samples to urine to identify the presence of leptospiral DNA. A total of 73 (68.9%) cows were infected, 64 of which (87.7%) were diagnosed via positive genital samples (uterine fragment and/or CVM), while only 14 (19.2%) by urine (*p* ≤ 0.001), reinforcing prior findings that testing genital samples, particularly CVM, is crucial to detect infected subfertile cows effectively [47].

Bovine cervical vaginal mucus (MCV) is a practical and valuable sample for field collection, as it reflects the uterine environment [11] and is an easy-to-collect sample. CVM can be used for direct detection of leptospiral DNA, enabling the identification of asymptomatic carriers and allowing targeted control measures to prevent the spread of the disease [11,26].

It is important to report that *sec*Y nucleotide sequencing identified all sequences as *L. interrogans*, with maximum identity (100%) with the *L. interrogans* strain UF24. This strain was originally identified in the uterine fragment of Brazilian cows with reproductive problems and is genetically close (>99%) to strains belonging to the Hardjoprajitno serovar from the Sejroe serogroup [48].

One main limitation of molecular diagnosis in animal leptospirosis is the absence of standardization. PCR-based tests have focused on both universal genes present in bacteria, such as *gyr*B, *rrs* (16S rRNA gene), and *sec*Y, as well as surface proteins restricted to *Leptospira*, such as *lip*L21, *lip*L32, *lip*L41, and *lig*B [49]. The *sec*Y gene is a housekeeping gene located on the CI chromosome that encodes a pre-protein translocase important for exporting proteins across the cytoplasmic membrane [50]. This gene has a good discriminatory power and gene sequence analysis, in addition to allowing the identification of species, and also provides a characterization of strains and, occasionally, genotypes. In this context, it is currently used in the second stage for discriminatory and taxonomic purposes, providing important epidemiological information [51]. In this study, all the *sec*Y sequences obtained were identical, emphasizing the presence of one major genotype associated with BGL, despite the differences regarding geographic regions of the herds. Recently, Borges and colleagues [52] showed that this sequence belongs to a haplotype group exclusively composed of genital strains. In light of these findings, efforts should be made to isolate strains of this genotype for better serological and molecular understanding, which could be an important step towards a specific BGL vaccine development.

Therefore, performing PCR after serology in herds that are reactive to *Leptospira* is crucial to improve diagnostic accuracy. Serology (MAT) may not necessarily indicate active infection, which is important for the proper management of bovine leptospirosis, especially since it is a chronic and silent disease. PCR, in turn, allows the detection of leptospiral DNA in several samples, identifying currently infected animals, even in the early stages when the immune response may not yet be sufficient to generate detectable antibodies [37]. Thus, the combination of these two approaches increases the sensitivity of the diagnosis and avoids underdiagnosis, allowing a better intervention in controlling the disease in cows [26]. Regarding the limitations of the study, it is worth highlighting the absence of a control group, which limits the comparison between animals exposed and not exposed to the pathogen. In addition, there was an unequal number of blood and CVM samples collected/received. Another important point is the low quantity of sequences obtained, which can be attributed to the loss of DNA during the purification stage, making it difficult to perform a more detailed analysis of the strains present. However, our results reinforce the importance of using a two-step protocol for diagnosing animals that do not present evident signs.

## 5. Conclusions

Bovine genital leptospirosis (BGL) is a silent but significant reproductive disease in cattle that leads to economic losses through infertility, repeated heat cycles with embryonic death, and abortions. It is commonly caused by strains of leptospires belonging to the Sejroe serogroup. Although serology (MAT) is widely used for screening at the herd level, it has limitations in identifying genital carriers, which can spread the disease without showing obvious symptoms.

In this study, we reinforce the use of a diagnostic protocol in animals with a history of reproductive diseases under field conditions, based on serology followed by PCR of CVM of the cows. Our results showed that this protocol was effective and practical for diagnosing BGL under field conditions. CVM sampling for PCR is simple and quick, making it a viable option for large-scale use. The combined MAT and CVM-PCR approach identified the carriers, enabling targeted treatment and better herd management. This protocol offers an efficient solution for detecting BGL, reducing its economic impact on cattle herds.

## Data Availability

The data that support the findings of this study are openly available in GenBank under accession numbers PQ039544-PQ039551.
